# Supplementary material for: Organophosphorus Flame Retardant TDCPP Displays Genotoxic and Carcinogenic Risks in Human Liver Cells
Source: Cells. 2022 Jan 7;11(2):195. doi: 10.3390/cells11020195 (PMC8773750; doi:10.3390/cells11020195)
Supplement: Supplementary file 1 [file cells-11-00195-s001.zip › cells-1523582-supplementary.pdf]

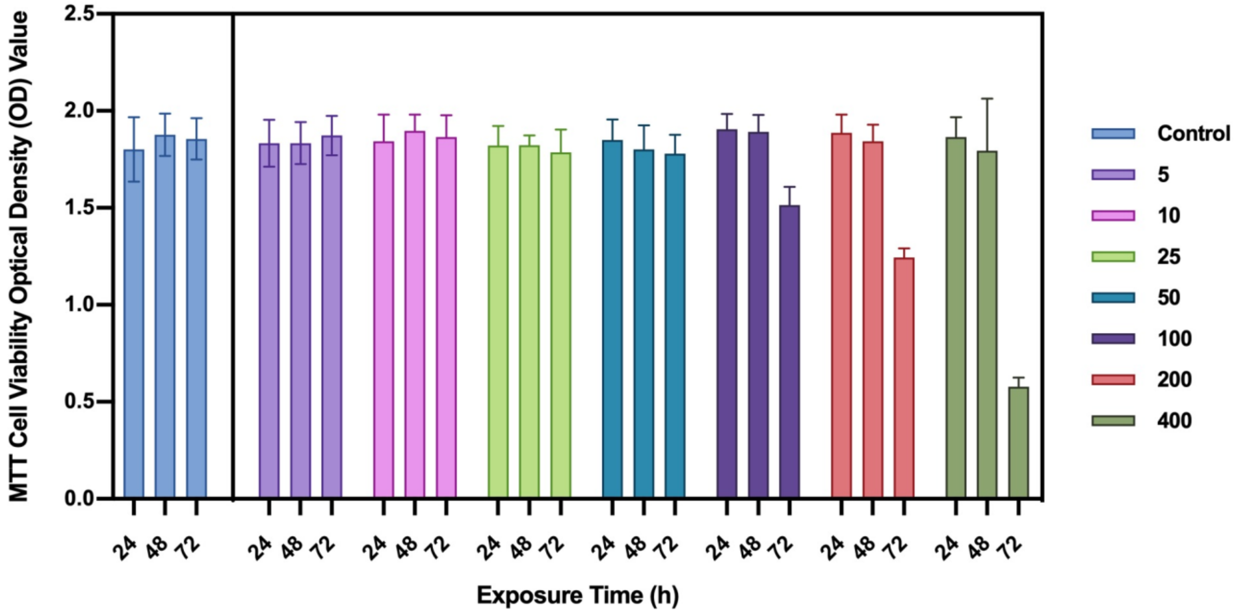

Supplementary Figure S1: Effect of TDCPP on HepG2 survival at different time points.

Supplementary Table S1. DNA damage analysis using comet assay parameters in TDCPP treated cells after 3 days of exposure.

Groups	Olive tail moment (Arbitrary Unit)	Tail length (µm)
Control	0.29 ± 0.05	46.35 ± 2.65
EMS (2 mM)	11.89 ± 1.92**	110.42 ± 6.83**
**TDCPP (µM)**		
100	1.90 ± 0.23**	54.62 ± 4.90**
200	4.81 ± 0.82**	72.08 ± 5.43**
400	8.87 ± 1.33**	114.56 ± 7.29**

Data represent the mean ± S.D. of three independent experiments done in duplicate. ***p*<0.01; EMS: Ethyl methanesulphonate.

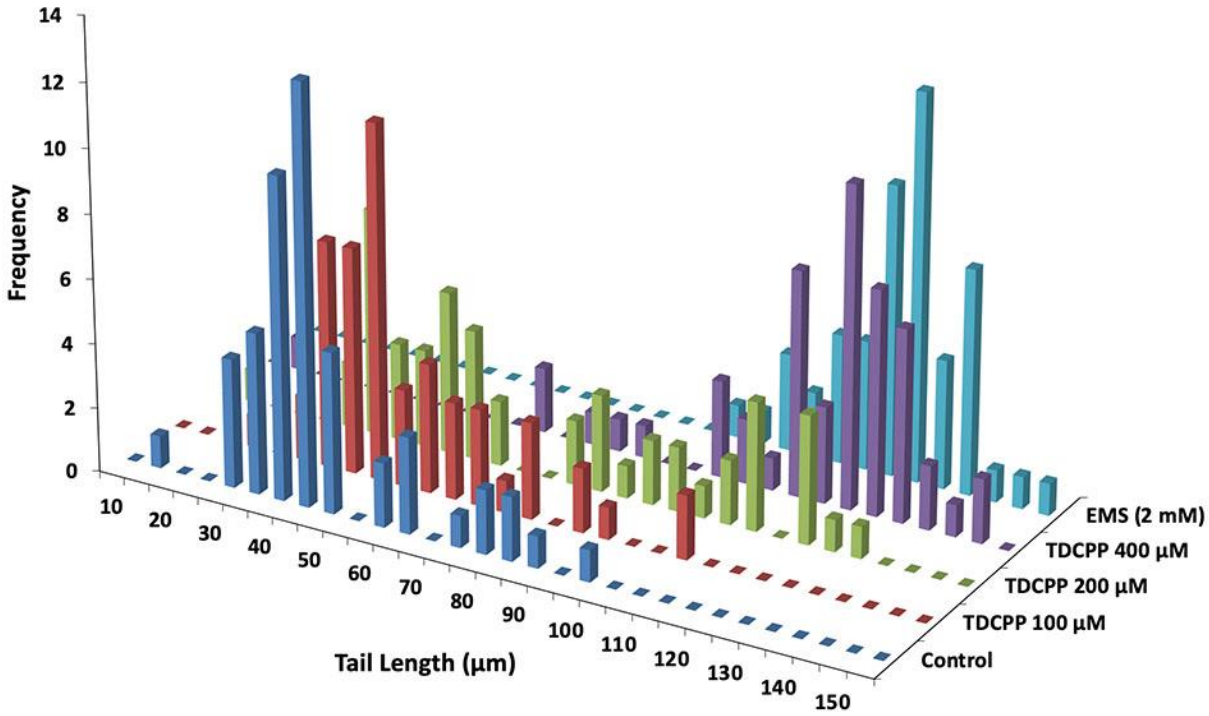

Supplementary Figure S2: Frequency distribution of DNA damage in TDCPP exposed cells.
